# 918. Developing “Words of Advice”: An Expressive Writing and Peer Support Intervention for Burn Survivors

**DOI:** 10.1093/jbcr/irag033.443

**Published:** 2026-04-06

**Authors:** Deanna C Denman, Elisa O'Hern, Alicia M Williams

**Affiliations:** US Army Institute of Surgical Research Burn Center, Joint Base San Antonio, Fort Sam Houston, TX; US Army, Potomac, MD; US Army Institute of Surgical Research Burn Center, Joint Base San Antonio, Fort Sam Houston, TX

## Abstract

**Introduction:**

Burn injuries carry a significant psychological burden, with survivors experiencing distress, isolation, and decreased quality of life following injury. This formative project explored the feasibility and thematic content of a brief, expressive writing intervention in which burn patients generated messages for future patients. The intervention aimed to promote psychological integration, support meaning-making, and create a bridge of peer support during early recovery.

**Methods:**

Following regulatory designation as not-research (preparatory project), 10 adult patients participated. Participants were invited to reflect on their experiences and generate advice intended for future patients facing similar injuries. Following the intervention, participants completed the Integration of Stressful Life Experiences Scale-Short Form (ISLES-SF) and provided qualitative feedback on the process. Written messages were thematically analyzed, with consensus determined by majority agreement (> 2 coders) to identify key emotional and cognitive themes.

**Results:**

All invited participants (10/10) completed the intervention and reported finding it meaningful. Participants ranged in their time since initial injury, but 9 out of 10 indicated they would have been open to completing the intervention while hospitalized. Message content revealed recurring themes of positivity and hope (8/10), grit and resilience (6/10), and connection through support of their family, friends, and the treatment team (4/10). Post-intervention ISLES-SF scores suggested moderately high narrative integration (m = 23; IQR = 21.25-24.75). Participants described the intervention as helpful and meaningful with several describing the personal satisfaction of contributing to others.

**Conclusions:**

This formative project suggests that a brief, message-generating intervention is both feasible, acceptable, and supportive of narrative integration among burn inpatients. Thematic analysis highlighted optimism, tenacity, and social connection. These results informed the refinement of a multi-session intervention now undergoing pilot testing and highlight the potential for broader integration of peer-oriented expressive writing into burn care.

**Applicability of Research to Practice:**

Expressive writing framed as peer support may facilitate narrative integration and meaning-making during early recovery.

**Funding for the study:**

N/A.

